# Compartmentalized cell envelope biosynthesis in *Mycobacterium tuberculosis*

**DOI:** 10.1128/mbio.02680-25

**Published:** 2025-11-26

**Authors:** Julia Puffal, Ian L. Sparks, James R. Brenner, Xuni Li, John D. Leszyk, Jennifer M. Hayashi, Scott A. Shaffer, Yasu S. Morita

**Affiliations:** 1 Department of Microbiology, University of Massachusetts Amherst 196202, Amherst, Massachusetts, USA; 2 Department of Biochemistry and Molecular Pharmacology, University of Massachusetts Medical School 356120, Worcester, Massachusetts, USA; 3 Mass Spectrometry Facility, University of Massachusetts Medical School 242978, Shrewsbury, Massachusetts, USA; Weill Cornell Medicine, New York, New York, USA

**Keywords:** cell envelope, cell membranes, glycolipids, lipid synthesis, membrane proteins, *Mycobacterium tuberculosis*

## Abstract

The inner membrane domain (IMD) is a metabolically active and laterally discrete membrane domain initially discovered in *Mycobacterium smegmatis*. The IMD correlates both temporally and spatially with the polar cell envelope elongation in *M. smegmatis*. Whether or not a similar membrane domain exists in pathogenic species remains unknown. Here, we show that the IMD is a conserved membrane structure found in *Mycobacterium tuberculosis*. We used two independent approaches, density gradient fractionation of membrane domains and visualization of IMD-associated proteins through fluorescence microscopy, to determine the characteristics of the plasma membrane compartmentalization in *M. tuberculosis*. Proteomic analysis revealed that the IMD is enriched in metabolic enzymes that are involved in the synthesis of conserved cell envelope components such as arabinogalactan and phosphatidylinositol mannosides. Using a fluorescent protein fusion of IMD-associated proteins, we demonstrated that this domain is concentrated in the subpolar region of the rod-shaped cells, where active cell envelope biosynthesis is taking place. Proteomic analysis further revealed the enrichment of enzymes involved in synthesis of phthiocerol dimycocerosates and phenolic glycolipids in the IMD. We validated the IMD association of two enzymes, α1,3-fucosyltransferase and fucosyl 4-*O*-methyltransferase, which are involved in the final maturation steps of phenolic glycolipid biosynthesis. Taken together, these data indicate that functional compartmentalization of membrane is an evolutionarily conserved feature found in both *M. tuberculosis* and *M. smegmatis*, and *M. tuberculosis* utilizes this membrane location to enrich biosynthetic enzymes for its surface-exposed lipid virulence factors.

**IMPORTANCE:**

*M. tuberculosis* remains an important public health threat, with more than one million deaths every year. The pathogen’s ability to survive in the human host for decades highlights the importance of understanding how this bacterium regulates and coordinates its metabolism, cell envelope elongation, and growth. The IMD is a membrane structure that associates with the subpolar growth zone of actively growing mycobacterial cell, but its existence is only known in a non-pathogenic model, *M. smegmatis*. Here, we demonstrated the presence of the IMD in *M. tuberculosis*, making the IMD an evolutionarily conserved plasma membrane compartment in mycobacteria. Furthermore, our study revealed that the IMD may function as the factory for synthesizing phenolic glycolipids, virulence factors produced by slow-growing pathogenic species.

## INTRODUCTION

Mycobacteria grow by depositing *de novo* synthesized cell envelope materials near the pole rather than the main sidewall of the rod-shaped cell (1–5). The cell envelope of mycobacteria is complex, composed of plasma membrane, peptidoglycan, arabinogalactan, and mycomembrane (6–9), making the spatially restricted cell envelope elongation a unique challenge for these bacteria. Precise coordination of the synthesis of cell envelope components is likely crucial, as highlighted by numerous proteins localizing to specific subcellular regions in mycobacteria (10). Among them, DivIVA, a filamentous coiled-coil protein, is associated with the pole of the cell, and its physical interaction with early cell elongation machinery is implicated in the determination of the site of cell envelope elongation (11, 12). The recruitment of other enzymes involved in cell envelope biosynthesis defines a subpolar growth zone where new envelope components are made and incorporated into the growing pole (5, 11, 13). Polar enrichment is observed not only for proteins but also for membrane lipids. Plasma membrane heterogeneity in mycobacteria was first reported using fluorescent lipid probes (14), and a more recent work suggested an enrichment of cardiolipin to the pole of growing mycobacterial cells (15).

Previously, we reported the presence of a growth-pole associated membrane domain, the inner membrane domain (IMD), in the nonpathogenic model organism *Mycobacterium smegmatis* (16–18). This domain is separated from the conventional plasma membrane upon density gradient fractionation of mycobacterial cell lysate. Plasma membrane is copurified with the cell wall, apparently due to physical connections between the two (termed PM-CW). The IMD, in contrast, is purified as ~50 nm membrane vesicles without the association of cell wall components. Among the proteins associated with the IMD, we find essential enzymes involved in lipid biosynthetic reactions. For instance, phosphatidylinositol mannosides (PIMs) are a major component of mycobacterial plasma membrane, and the initial phase of their biosynthesis is enriched in the IMD (16, 17). The function of the IMD is not limited to the biosynthesis of the cell envelope. For example, menaquinone is a major respiratory chain electron carrier in mycobacteria, and enzymes that mediate the final maturation steps of its biosynthesis are enriched in the IMD (19). These observations indicate the diversity of crucial processes required for polar cell envelope growth and central metabolism that are associated with, and possibly coordinated within, this membrane domain.

In *Mycobacterium tuberculosis*, while comparative proteomic analyses of secreted and envelope-associated proteins have been reported (20, 21), we know comparatively less about lateral distribution of plasma membrane-associated proteins. DivIVA is associated with the poles in *M. tuberculosis* (11) and CwsA, which plays a role in cell wall synthesis and interacts with DivIVA, also shows polar and mid-cell localization (22). On the other hand, FtsZ and its interacting partner SepF localize to future division sites (23, 24). Furthermore, the sensor kinase MtrB localizes to septa in *M. tuberculosis* as well (25). These past studies show conserved features of pole- and septum-associated membrane proteins. However, other types of membrane compartmentalization have not been reported in pathogenic mycobacteria. In this study, we investigated the presence of the IMD in *M. tuberculosis*. Using subcellular fractionation, we first examined the IMD proteome in comparison to the PM-CW proteome. We then visualized the localization of the IMD in live growing *M. tuberculosis*. Our results demonstrate that the IMD is a membrane domain conserved in mycobacteria.

## RESULTS

### Density gradient fractionation of the *M*. *tuberculosis* cell lysate

**Fig 1:**
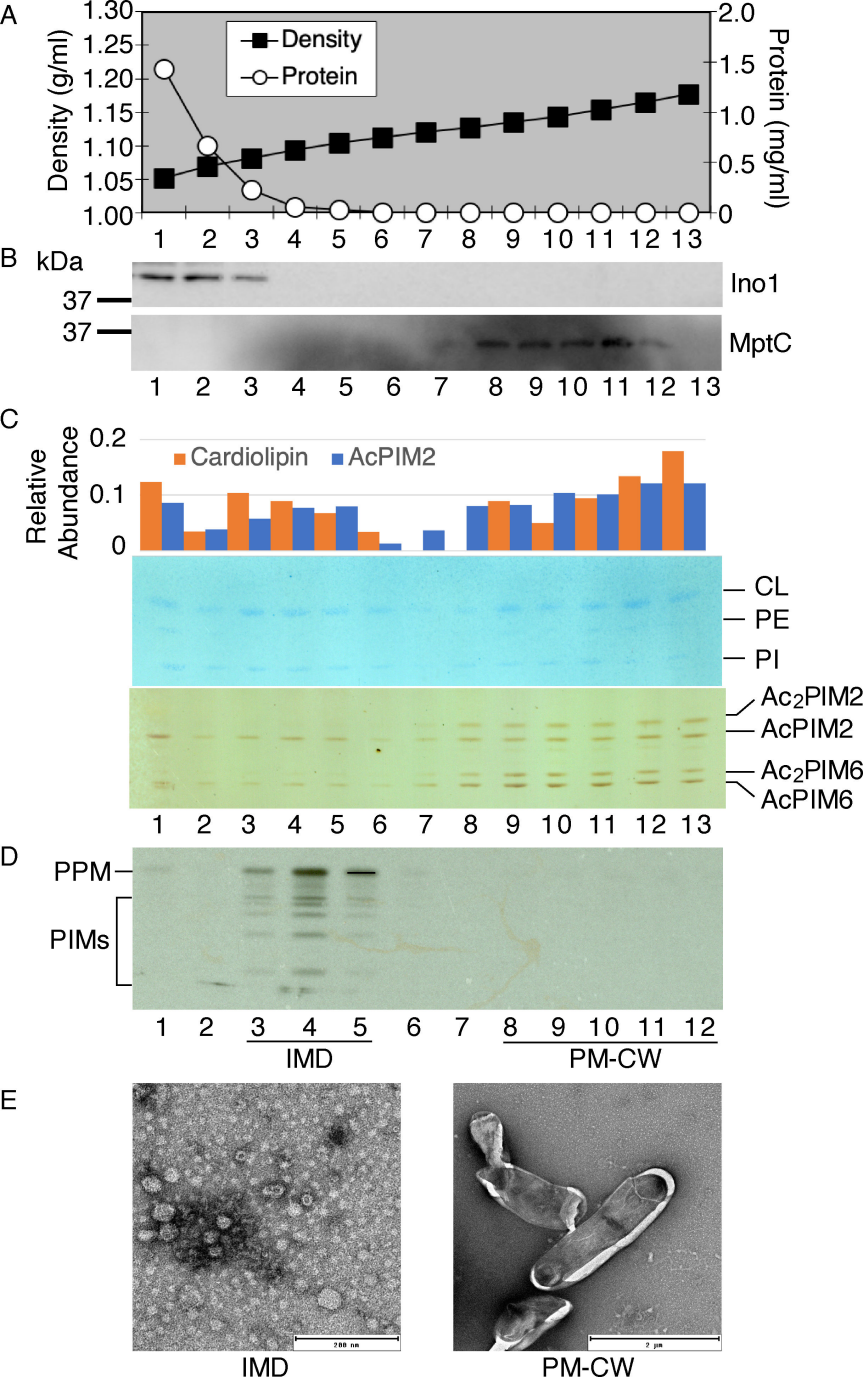
Membrane compartmentalization in *M. tuberculosis* mc^2^6230. (**A**) The sucrose density and protein concentration of the gradient fractions. Soluble cytoplasmic proteins are dominant, making total protein concentration reflective of cytoplasmic fractions. IMD (fractions 3–5), 1.08–1.11 g/mL; PM-CW (fractions 8–12), 1.13–1.17 g/mL. (**B**) Immunoblot detection of cytoplasmic marker Ino1 (40 kDa) and PM-CW marker MptC (47 kDa). (**C**) Distribution of membrane phospholipids across the gradient fractions. Phospholipids were stained by molybdenum blue staining. PIMs were detected by orcinol staining. The graph indicates densitometric measurements of cardiolipin from molybdenum blue staining and AcPIM2 from orcinol staining. Note that lipids detected in the top fraction of the density gradient fractions are reminiscent of a previous observation in the fractionation of *M. smegmatis* cell lysate (17) and could be attributed to the flotation of lipid droplets (27). (**D**) Cell-free PPM biosynthesis detection by GDP-[^3^H]-Mannose radiolabeling, indicating active PPM synthesis in the IMD fractions. (**E**). Negative staining EM of the IMD and PM-CW fractions.

We prepared a lysate of exponentially growing *M. tuberculosis* cells and fractionated by density gradient sedimentation (Fig. 1A). As previously seen in *M. smegmatis*, the majority of proteins were enriched in the top two fractions. In the density gradient of *M. smegmatis* lysate, cytoplasmic proteins are retained in the top fractions and do not sediment significantly. To confirm that the *M. tuberculosis* proteins in the top fractions correspond to cytoplasmic proteins, we performed immunoblotting against the cytoplasmic enzyme Ino1, an inositol-3-phosphate synthetase (26), and detected this protein predominantly in fractions 1 and 2 (Fig. 1B). Next, we determined the localization of the mannosyltransferase MptC, a polytopic membrane protein found in the PM-CW in *M. smegmatis*, by immunoblotting. MptC was enriched in the denser region of the gradient, spanning from fractions 8–11 (Fig. 1B). These fractions correspond to the density of 1.127–1.154 g/mL, which is comparable to 1.131–1.159 g/mL for *M. smegmatis* PM-CW (19), suggesting that MptC is a PM-CW protein in *M. tuberculosis* as well.

To examine if the IMD exists in *M. tuberculosis* in addition to the PM-CW fraction, we examined the compositional profiles of plasma membrane phospholipids. Lipids were extracted from each fraction and visualized by chemical staining. Due to low yields of lipids from density gradient fractions of *M. tuberculosis* lysates, the intensity levels of some lipids such as phosphatidylethanolamine (PE) and phosphatidylinositol (PI) were near the limit of detection (Fig. 1C). In contrast, because glycolipid detection by orcinol is more sensitive than phospholipid detection by molybdenum blue, we were able to visualize PIMs more clearly even though PIMs are less abundant than other phospholipids. We quantified the intensity profiles of cardiolipin (CL) and AcPIM2 across the gradient fractions using the quantification method described previously (28). Both CL and AcPIM2 were detected in the PM-CW fractions as well as in another density region spanning from fractions 3 to 5. We additionally quantified PE by positive ion LC-MS (see Fig. S1 at https://doi.org/10.5281/zenodo.17547108). Lipid extracts from density gradient fractions were purified by chloroform/methanol extraction followed by butanol/water partitioning. Each extract was spiked with an equal amount of PI C8:0, a non-native lipid standard for normalizing LC-MS peak intensities across samples. Fractions 3–6, corresponding to the IMD, contained 21% of the total PE 34:1 and 16% of the total PE 35:0, while the PM-CW (fractions 8–12) contained 66% of the total PE 34:1 and 82% of the total PE 35:0 (Fig. S1). These data suggest that there is an additional membrane fraction separate from the PM-CW. One known reaction that takes place in the *M. smegmatis* IMD is the biosynthesis of polyprenol-phosphate-mannose (PPM). We have previously shown that the PPM biosynthetic activity is enriched in the IMD and the enzyme Ppm1 was found exclusively in the IMD in *M. smegmatis* (16, 17). To determine the localization of this biosynthetic reaction in *M. tuberculosis*, we performed a cell-free radiolabeling assay using GDP-[^3^H]mannose as a mannose donor. As shown previously (29–31), *M. smegmatis* produces heptapentenyl and decapentenyl PPM species (see Fig. S2 at https://doi.org/10.5281/zenodo.17547108). In contrast, *M. tuberculosis* does not produce heptapentenyl polyprenols, and decapentenyl PPM is the major product (31–33). Although the enzyme activity is not as robust as that of *M. smegmatis* enzyme, we detected the synthesis of the expected decapentenyl PPM from *M. tuberculosis* cell lysate (Fig. S2), and was enriched in fractions 3-5 of a sucrose density gradient (Fig. 1D), suggesting the subcellular localization of PPM synthetase in these membrane fractions.

To compare the morphology of the membrane present in fractions 3–5 with that of the PM-CW, we precipitated these membrane fractions by differential centrifugation and visualized them by negative staining electron microscopy (EM) (Fig. 1E). As in *M. smegmatis* (17), we observed small vesicle-like structures in fractions 3–5, while the PM-CW fraction revealed larger fragments of cells. Together, these data support that fractions 3–5 correspond to the IMD in *M. tuberculosis* and its overall characteristics are similar to those of the *M. smegmatis* IMD.

### Identification of IMD-associated proteins in *M. tuberculosis*

**Fig 2:**
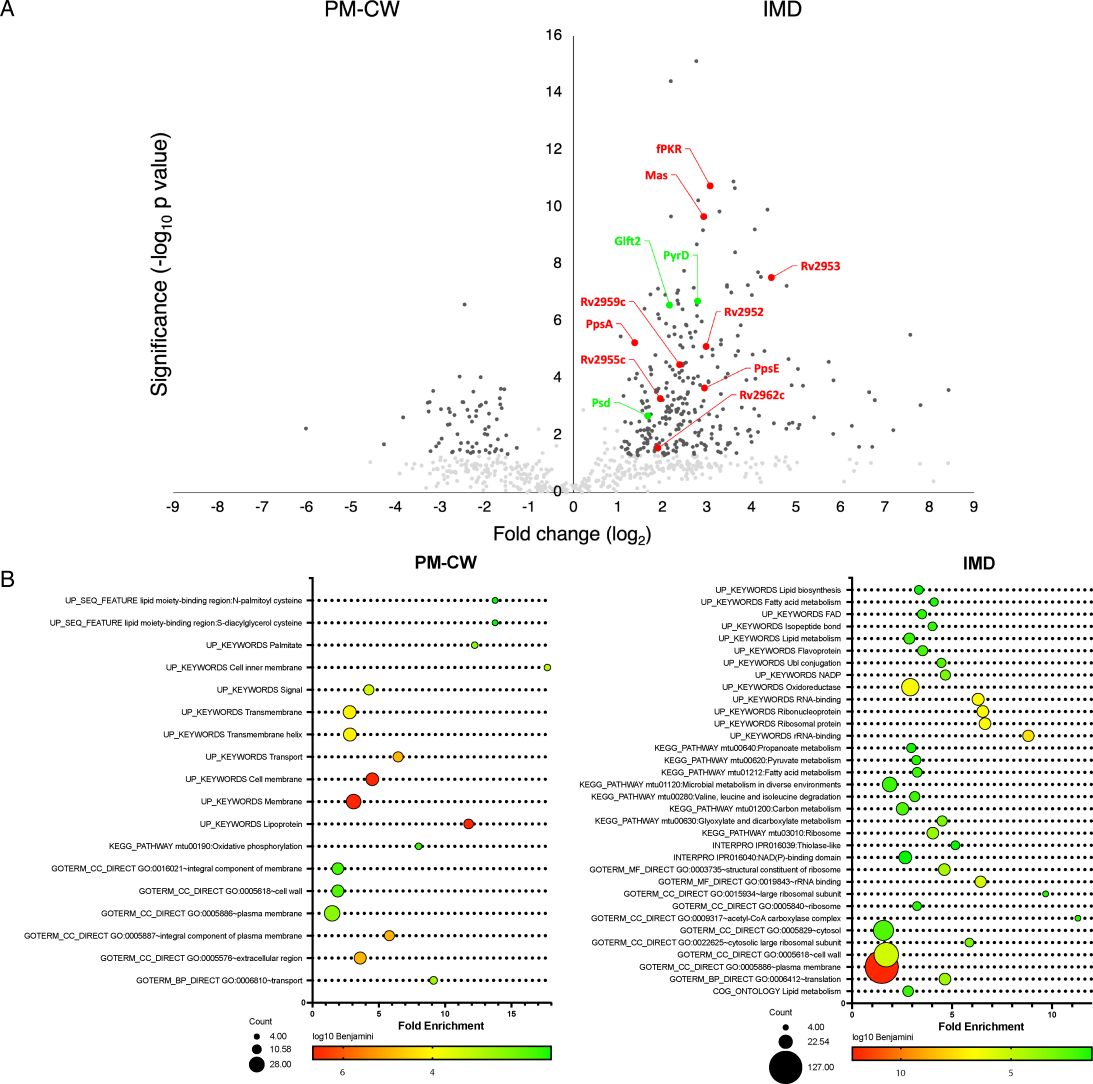
Comparative proteome of the conventional plasma membrane (PM-CW) and the IMD. Cell growth, lysate preparation, membrane purification and proteomic analysis were repeated three times. (**A**) The volcano plot of proteins enriched in either the PM-CW or the IMD. Proteins that were enriched more than twofold with *P* < 0.05 were considered significant (black dots). Other identified proteins are shown in gray dots. Red dots correspond to significantly enriched proteins involved in PGL/PDIM biosynthesis. Green dots correspond to other proteins analyzed biochemically and/or by fluorescent microscopy in this study. (**B**) Functional annotation of proteins enriched in the membrane fractions using UniProt keywords, showing that the IMD is enriched in proteins related to membrane and lipid metabolism. The enrichment represents the proportion of the term in the IMD proteome compared to its expected proportion in the genome. The areas of the circles are proportional to the number of proteins in each category.

To uncover the IMD proteome, we purified the membranes in both IMD and PM-CW fractions by differential centrifugation and analyzed the trypsin digests of total proteins by liquid chromatography-mass spectrometry (LC-MS) in biological triplicates. The comparative proteomes of the two membranes were distinct with a total of 64 and 298 proteins enriched more than twofold (*P* < 0.05, Mann-Whitney test) in the PM-CW or the IMD, respectively (Fig. 2A, also see Data set S1 at https://doi.org/10.5281/zenodo.17547108). PM-CW was associated with enzymes with activities that are related to the typical functions of plasma membrane (Fig. 2B). For example, we identified ESX-5 type VII secretion system components EccB5-E5 (Rv1782, Rv1783, Rv1795), trehalose monomycolate transporter MmpL3 (Rv0206c), a subunit of the phosphate specific transport system, PstS1 (Rv0934), and NADH-quinone oxidoreductase subunits NuoF and NuoG (Rv3150, Rv3151). The IMD, on the other hand, was more restricted to enzymes involved in lipid metabolism (Fig. 2B). For instance, we found enzymes involved in the synthesis of polyprenol-linked galactan precursor for arabinogalactan synthesis enriched in the IMD: the GlcNAc-diphospho-decaprenol L-rhamnosyltransferase WbbL1 (Rv3265c), the Rha-GlcNAc-diphospho-decaprenol β1,4/1,5-galactofuranosyltransferase GlfT1 (Rv3782), and the Gal*f*-Gal*f*-Rha-GlcNAc-diphospho-decaprenol β1,5/1,6-galactofuranosyltransferase GlfT2 (Rv3808c). The PPM synthase Ppm1 (Rv2051c) and acyl-CoA dehydrogenases such as FadE24 (Rv3139), FadE23 (Rv3140), and FadE10 (Rv0873) were also found in the IMD. From the total of 298 proteins identified in the IMD through the comparative proteomics, 218 had *M. smegmatis* homologs. Of these, 101 have also been identified previously as enriched in the IMD in a comparative proteomics of the *M. smegmatis* IMD and PM-CW (Data set S1) (16). These data are consistent with the idea that the *M. tuberculosis* IMD is a metabolically active membrane domain with multiple functions shared with that of *M. smegmatis*.

### Validation of IMD-associated proteins

**Fig 3:**
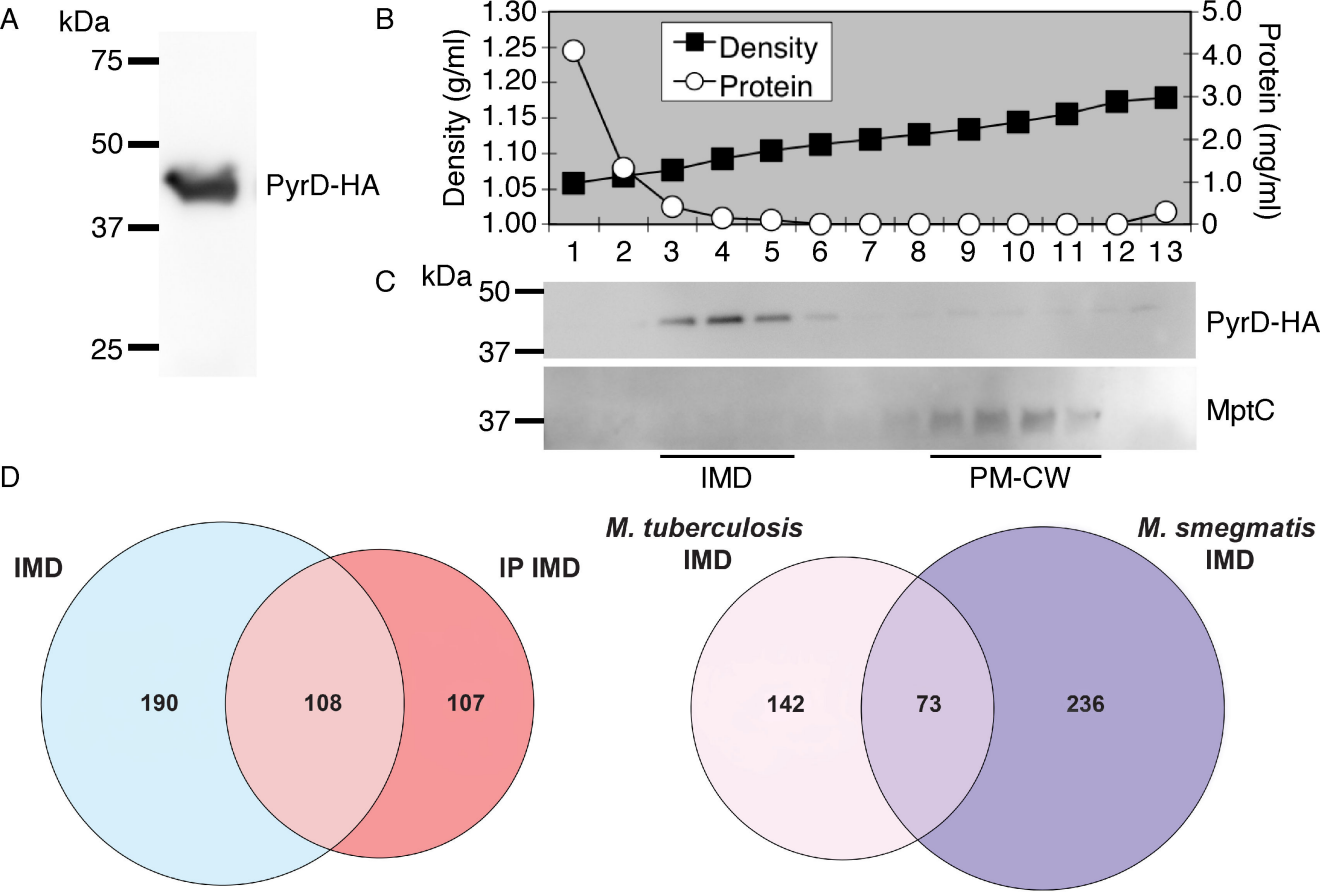
PyrD is an IMD-associated protein. (**A**) Immunoblotting of a crude lysate prepared from a merodiploid *M. tuberculosis* strain producing PyrD-HA. In this strain, the expression vector was integrated at L5 *attB* site. Expected molecular weight of PyrD-HA is 42.1 kDa. (**B**) Sucrose density gradient fractionation. The density and protein concentration for each fraction are shown. IMD (fractions 3–5), 1.08–1.11 g/mL; PM-CW (fractions 9–12), 1.13–1.17 g/mL. (**C**) Immunoblot detection of PyrD-HA in the IMD fractions and the PM-CW marker MptC (Rv2181, 47 kDa). (**D**) Proteome of an immunoprecipitated IMD. The IMD was purified from the PyrD-HA-expressing strain using anti-HA antibody. Left, Venn diagram showing the overlap of proteins found in the comparative IMD proteome (Fig. 2) and the immunoprecipitated IMD proteome. Right, Venn diagram showing the orthologs found commonly in the immunoprecipitated IMD proteomes of *M. tuberculosis* and *M. smegmatis*.

As the IMD sediments slower than PM-CW in a sucrose gradient, the IMD fraction may overlap with the cytoplasmic fraction and contain cytoplasmic proteins. Therefore, it is valuable to validate the proteome data set using alternative approaches. Among the IMD-associated proteins was the dihydroorotate dehydrogenase PyrD, which is involved in pyrimidine biosynthesis and is dependent on a lipidic electron carrier, menaquinone, for the redox reaction (34, 35). Because it is an IMD-associated protein in *M. smegmatis* (16), we chose this protein for the validation of the *M. tuberculosis* IMD proteome. We cloned the gene with a hemagglutinin (HA) epitope tag into an expression vector, integrated into the bacteriophage L5 integration site of *M. tuberculosis* genome, and confirmed its expected molecular weight of 42.1 kDa (Fig. 3A). We then fractionated the cell lysate by sucrose density gradient (Fig. 3B) and confirmed the IMD localization of PyrD-HA in contrast to the PM-CW localization of MptC (Fig. 3C). These data indicate that PyrD associates with the IMD in *M. tuberculosis* validating the comparative proteomics analysis.

Next, we took advantage of the PyrD-HA-expressing strain and purified the IMD from the gradient fractions by vesicle immunoprecipitation (IP) using anti-HA agarose beads as performed previously in *M. smegmatis* (16). We used the IMD fraction from the parental strain as a negative control for immunoprecipitation. Unlike the comparative proteomics shown in Fig. 2, proteomic analysis of these immunoprecipitated samples does not reveal relative enrichment of proteins between the IMD and PM-CW fractions. However, it can remove co-fractionating proteins that are not physically bound to the IMD and identify IMD-associated proteins that are not enriched in the IMD compared with the PM-CW. With a cutoff of fivefold enrichment between the experimental and negative control samples, 215 proteins were identified in the IMD of *M. tuberculosis* (see Data set S1, IMD [IP], at https://doi.org/10.5281/zenodo.17547108). Combined with 298 proteins identified as IMD-enriched relative to PM-CW (Data set S1, IMD), there are 404 proteins that were labeled as IMD proteins (Data set S1, IMD [All]). Among them, 108 proteins were found in both IMD proteomes (Data set S1, IMD [Both]) (Fig. 3D). Among the remaining 107 immunoprecipitated proteins that were not found in the comparative IMD proteome, 15 were found enriched in the PM-CW and 23 were found in both fractions (Data set S1, PM-CW and IMD [IP]), suggesting that some proteins can associate with both membrane fractions. Out of the 215 immunoprecipitated proteins, 148 had *M. smegmatis* homologs, with 73 localizing in the IMD of both species (Fig. 3D). Sixty-seven IMD-associated proteins were unique to *M. tuberculosis*, and notable proteins among them were enzymes involved in the biosynthesis of phthiocerol dimycocerosate (PDIM) and phenolic glycolipids (PGL; see below for further analysis). These data indicate that the IMD is an evolutionarily conserved domain in mycobacteria with species-specific features.

### Visualization of IMD proteins in *M. tuberculosis*

**Fig 4:**
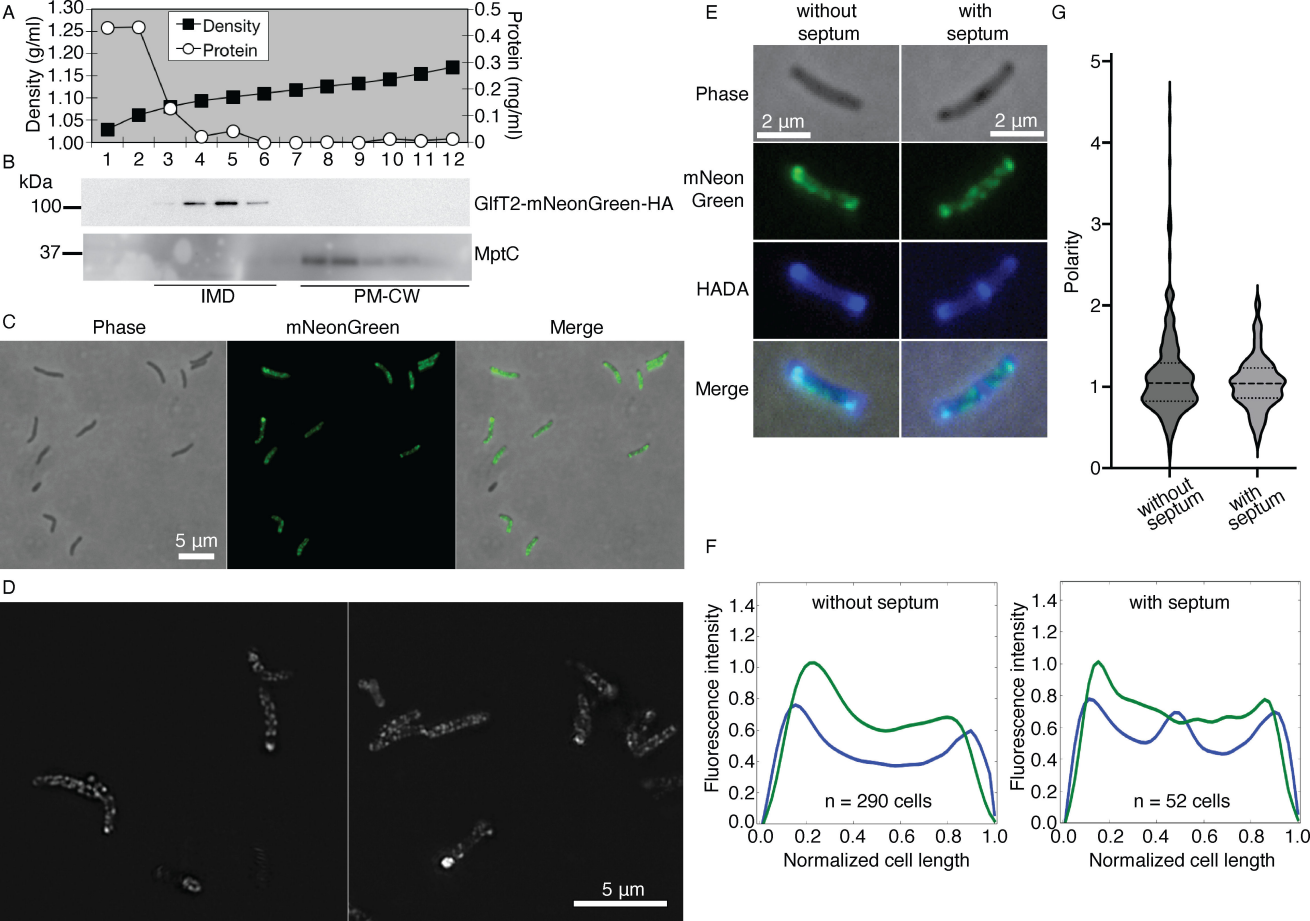
Polar enrichment and sidewall patches of GlfT2-mNeonGreen-HA, an IMD-associated protein. (**A**) Density gradient and protein concentration profiles. IMD (fractions 3–6), 1.08–1.11 g/mL; PM-CW (fractions 8–12), 1.13–1.17 g/mL. (**B**) Immunoblotting of GlfT2-mNeonGreen-HA (100.9 kDa). MptC, PM-CW marker. (**C**) Conventional fluorescence microscopy images of log phase cells. (**D**) SIM images of log phase cells. (**E**) Cells expressing GlfT2-mNeonGreen-HA, labeled with HADA for 2 h and visualized by conventional fluorescence microscopy. (**F**) Fluorescence intensity profile of GlfT2-mNeonGreen-HA from cells labeled with HADA, for which representative images are shown in panel **E**. Cells were categorized into two populations based on the presence or absence of a septum, as determined by HADA labeling. Intensity profiles are aligned with the brightest pole on the left and are shown in arbitrary units. Green and blue fluorescence intensity profiles correspond to GlfT2-mNeonGreen-HA and HADA, respectively. (**G**) Violin plots showing the distribution of the polarity in individual cells. The bright pole was defined as the brighter of the two 10-pixel (0.74 µm) regions at each end of the cell. Polarity was calculated as the mean fluorescence of the bright pole divided by the mean fluorescence of the remainder of the cell. The plots were generated from 290 non-septated and 52 septated cells. Experiments are repeated at least twice and representative results are shown.

We constructed a strain, in which the expression of PyrD-2×HA-tagRFP is driven by the endogenous promoter. We grew the strain, fractionated the cell lysate by sucrose density gradient, and confirmed the IMD localization of PyrD by immunoblotting (see Fig. S3 at https://doi.org/10.5281/zenodo.17547108). While this strain was useful for confirming the IMD localization of PyrD through a fusion protein expressed from the endogenous locus, we could not visualize its localization by fluorescence microscopy (not shown). As an alternative approach, we used an expression vector that integrates at the mycobacteriophage L5 *attB* site and expressed IMD-associated proteins as fluorescent protein fusions using a strong promoter. We chose two proteins: GlfT2, a galactosyltransferase involved in galactan biosynthesis, and Psd, a phosphatidylserine decarboxylase involved in PE biosynthesis (Fig. 2A). These enzymes are also enriched in the IMD of *M. smegmatis* (16, 17). We fractionated the lysates of cells expressing either GlfT2-mNeonGreen-HA or Psd-mNeonGreen-HA. Both GlfT2-mNeonGreen-HA and Psd-mNeonGreen-HA showed expected molecular weights (100.9 and 53.5 kDa, respectively) and were enriched in the IMD fractions (Fig. 4A and B; see Fig. S4A and B at https://doi.org/10.5281/zenodo.17547108) although we noted that there was a weak signal of Psd-mNeonGreen-HA in the PM-CW.

We then analyzed the localization of these proteins in live cells by fluorescence microscopy. In a subset of cells, GlfT2-mNeonGreen-HA showed intense fluorescence near the poles of the cells with less intense patches of fluorescence along the sidewall membrane (Fig. 4C). This fluorescence pattern is consistent with the IMD fluorescence patterns observed in *M. smegmatis*. We used structured illumination microscopy (SIM) to acquire higher-resolution images. As shown in Fig. 4D, intense foci near the poles of the cells were evident and the sidewall patches appeared consistent with the localization of a membrane-associated protein. Even though the subpolar enrichment of GlfT2-mNeonGreen-HA was evident in some cells, we often found many cells where the intensities of subpolar and sidewall patches were similar, making the subpolar enrichment not as obvious as in the case of *M. smegmatis*. We considered the possibility that the subpolar IMD enrichment may correlate with a specific cell cycle stage. To visualize cells with or without active septum synthesis, we labeled the cells with 3-[[(7-hydroxy-2-oxo-2H-1-benzopyran-3-yl)carbonyl]amino]-d-alanine (HADA), a fluorescent d-amino acid analog (Fig. 4E), and separated the cell population based on the presence of a septum. We then determined the average fluorescence intensity profiles of GlfT2-mNeonGreen-HA along the cell length as we have previously done in *M. smegmatis* (36). We observed that the enrichment of GlfT2-mNeonGreen-HA was slightly proximal to the polar HADA labeling and was evident regardless of whether there was an active septum synthesis or not (Fig. 4F). To calculate the subpolar enrichment of individual cells, we determined the average intensity of the bright pole and divided it by the average intensity of the rest of the cell. We plotted the polarity of individual cells that are categorized into septated and non-septated cells (Fig. 4G). In both septated and non-septated populations, about half (51.7% and 53.8%, respectively) of the cells showed polar enrichment (polarity > 1). However, the remaining halves did not show subpolar enrichment, regardless of whether cells are dividing, suggesting substantial heterogeneity even in logarithmically growing cell populations. Additionally, we found cells with a high polarity value of 2 or greater more frequently in non-septated cells (6.2%) than in septated cells (1.9%). Comparable fluorescence patterns were also observed for Psd-mNeonGreen-HA (see Fig. S4C at https://doi.org/10.5281/zenodo.17547108). Together, these data show that the IMD is a membrane domain that can be enriched in the subpolar region of the cell.

### Tuberculosis virulence factors mature in the IMD

**Fig 5:**
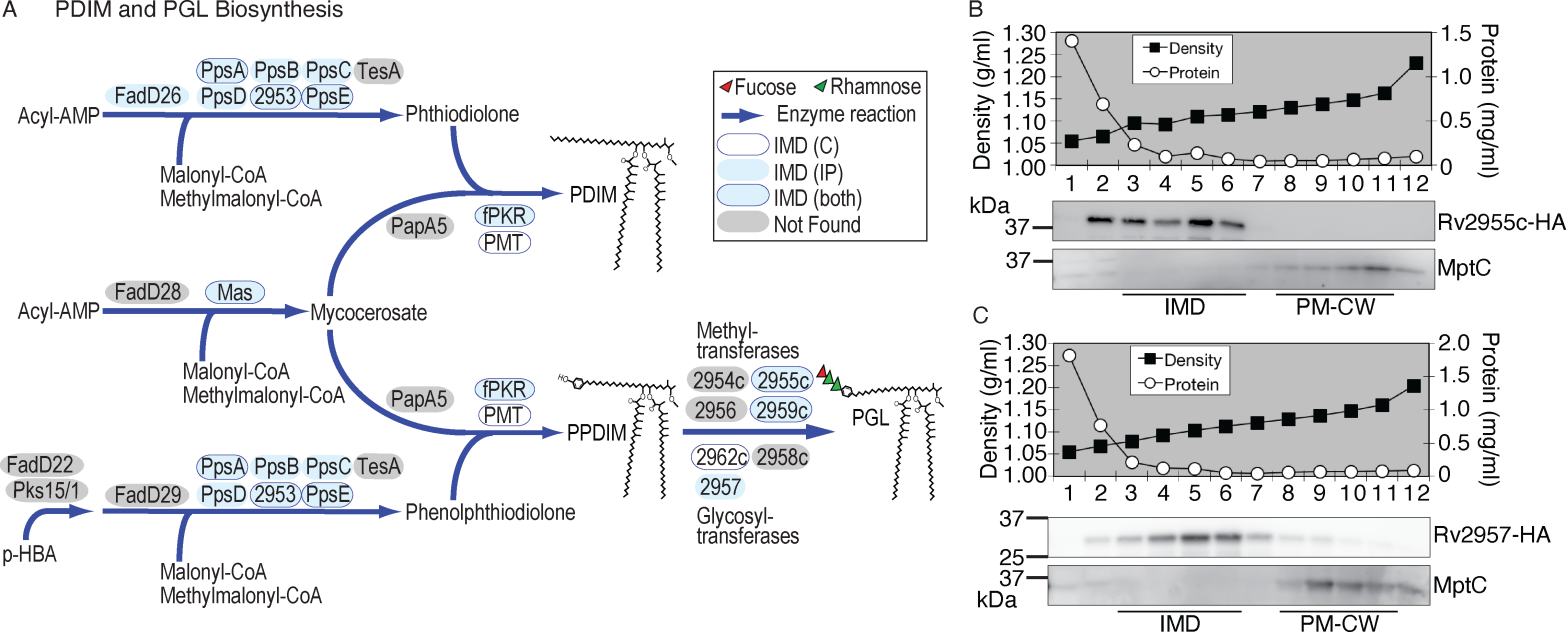
The biosynthetic enzymes of lipid virulence factors are enriched in the IMD. (**A**) Biosynthetic pathways of PDIM and PGL. Enzymes involved in the biosyntheses are shown in ovals, which are color-coded to indicate the subcellular localization based on proteomic analyses. (**B**) Subcellular fractionation of *M. tuberculosis* cells expressing Rv2955c-HA. Profiles of sucrose density gradient, total protein concentration, and immunoblotting of Rv2955c-HA (37.8 kDa). IMD (fractions 3–6), 1.10–1.11 g/mL; PM-CW (fractions 8–11), 1.13–1.16 g/mL. (**C**) Subcellular fractionation of *M. tuberculosis* cells expressing Rv2957-HA. Profiles of sucrose density gradient, total protein concentration, and immunoblotting of Rv2957-HA (32.9 kDa). IMD (fractions 3–6), 1.08–1.11 g/mL; PM-CW (fractions 8–11), 1.13–1.16 g/mL. MptC, PM-CW marker.

The biosynthesis pathways of PDIM and PGL, mycomembrane-associated lipid virulence factors, are partially overlapping, utilizing the same enzymes for several steps (Fig. 5A). The pathway can be divided into synthesis, assembly, and maturation stages. In the synthesis stage, mycocerosates and (phenol)phthiocerols are synthesized by Mas and PpsA-E, respectively. In the assembly stage, PapA5 esterifies two molecules of mycocerosates to the hydroxyl groups of phthiocerol or phenolphthiocerol, producing PDIM or a PGL precursor, phenolphthiocerol dimycocerosate (PPDIM), respectively. In the maturation stage, PPDIM is further decorated at the hydroxyl group of the phenol moiety by several carbohydrate residues, and these carbohydrates are modified by methylation to become a mature PGL.

We found several enzymes in these pathways enriched in the IMD (Fig. 5A). We generated merodiploid strains producing HA-tagged enzymes involved in the final maturation steps of PGL synthesis, namely a rhamnosyl *O*-methyltransferase (Rv2959c), a fucosyltransferase (Rv2957), and a fucosyl *O*-methyltransferase (Rv2955c). All three enzymes were enriched in the immunoprecipitated IMD. Rv2955c and Rv2959c were also enriched in the IMD based on the IMD/PM-CW comparison. We generated merodiploid strains producing fusion proteins tagged with a short HA epitope alone. Among them, we detected a protein band that migrated near the expected molecular weight for Rv2955c and Rv2957 (Fig. 5B and C). We fractionated a cell lysate from the strain producing either Rv2955c-HA or Rv2957-HA and determined the subcellular localization. Both proteins were localized to the IMD although Rv2955c was distributed more broadly to a lighter density region as well (Fig. 5B and C). Thus, our biochemical analysis validates that enzymes that mediate the final maturation steps of PGL synthesis are enriched in the IMD. Taken together with proteomic identifications, our data support the idea that the majority of enzymatic reactions to synthesize PDIM/PGL are localized to the IMD.

## DISCUSSION

The IMD is proposed to provide a membrane surface for the cell envelope biosynthesis and growth of *M. smegmatis*, a non-pathogenic model *Mycobacterium* (16, 17). Here, we demonstrated that a similar membrane organization exists in *M. tuberculosis*. Furthermore, our data suggest that the biosynthesis of PGL, a virulence factor produced by a subset of pathogenic mycobacteria (37–41), takes place in the IMD. We propose that the IMD is an evolutionary conserved subcellular organization in mycobacteria.

A number of metabolic reactions in the IMD are conserved between the two species. From the 108 proteins detected in both comparative and IP proteomics, 69 had *M. smegmatis* orthologs. From these, 54 were present in the IMD of both mycobacteria species. The first example is GlfT2, a galactofuranosyltransferase involved in arabinogalactan synthesis, which was found in the IMD of both species (16). Two reactions preceding the GlfT2-driven galactose chain elongation are mediated by the rhamnosyltransferase WbbL1 (Rv3265c) and the galactofuranosyltransferase GlfT1 (Rv3782). Both WbbL1 and GlfT1 are enriched in the IMD proteome of *M. smegmatis* as well as *M. tuberculosis* (16). While enzyme localizations may not necessarily indicate the active site of galactan biosynthesis (42), our data imply conserved function of the IMD. The second example is polyprenol-phosphate-mannose biosynthesis, which is IMD-associated in *M. smegmatis* and the enzyme Ppm1 is enriched in this membrane domain (16, 17). In *M. tuberculosis*, we used radiolabeled GDP-mannose to show the enzyme activity of Ppm1 in the IMD. Furthermore, we detected Ppm1 (Rv2051c) in the *M. tuberculosis* comparative IMD proteome although we could not detect this protein from the immunoprecipitated IMD. In *M. smegmatis*, the PPM synthase Ppm1 and the integral membrane protein Ppm2 are produced separately, while they are produced as a single fused polypeptide in *M. tuberculosis* (43). The larger and more hydrophobic nature of *M. tuberculosis* Ppm1 may have contributed to the inefficient detection of this protein by proteomics. Finally, PyrD, a menaquinone-dependent dihydroorotate dehydrogenase involved in pyrimidine biosynthesis, is an IMD-associated protein in *M. smegmatis* (16, 27), and our current study revealed it as an IMD protein in *M. tuberculosis* as well. These pathways represent some of the well-characterized evolutionarily conserved pathways enriched in the IMD. In total, our data showed that 69%–73% of the IMD-associated *M. tuberculosis* proteins had *M. smegmatis* orthologs, which was comparable to the two-species overlap found in the whole genome, where 63%–69% of *M. tuberculosis* protein-coding genes have *M. smegmatis* orthologs (44, 45).

Only two proteins detected in the IMD of *M. tuberculosis* were previously described as PM-CW associated proteins in *M. smegmatis*. Among them, the localization of Pks13 (Rv3800c) to the IMD in *M. tuberculosis* could indicate the final step of mycolic acid synthesis takes place in this membrane (46). This localization is generally consistent with previous findings showing the mycomembrane and mycolic acid assembly associated with the poles of mycobacteria (10). However, no other protein associated with this pathway was detected in the present study or in the IMD of *M. smegmatis*, underscoring the need for additional experimental verifications.

It is notable that the IMD is often where final maturation steps of lipid biosynthesis take place. For example, the final step of PE biosynthesis is decarboxylation of phosphatidylserine mediated by the IMD-associated enzyme Psd (17, 27). We confirmed in this study that Psd (Rv0437c), which was expressed as a mNeonGreen-HA fusion protein, is an IMD-associated protein by both subcellular fractionation and fluorescence microscopy. Similarly, our data suggest that during the synthesis of an electron carrier, menaquinone, demethylmenaquinone produced by the PM-CW enzyme MenA is modified by the IMD-associated demethylmenaquinone methyltransferase MenG and menaquinone reductase MenJ to become a mature form of menaquinone (19). We identified both MenG (Rv0558) and MenJ (Rv0561c) in the proteome of the immunoprecipitated *M. tuberculosis* IMD, indicating a conserved compartmentalization of these final two steps of menaquinone biosynthesis in the pathogenic species.

In addition to these shared features, the *M. tuberculosis* IMD revealed roles that are specific to slow-growing pathogens and not found in *M. smegmatis*. In particular, we found multiple enzymes associated with the PDIM/PGL biosynthesis. These lipid virulence factors are not produced by *M. smegmatis*, and genes encoding the biosynthetic enzymes are absent in its genome. We found PpsA–E (Rv2931–2935), polyketide synthases that produce phthiodiolone and phenolphthiodiolone, enriched in the IMD proteomes. They are large proteins, ranging 159–231 kDa, and therefore, we were initially concerned if the IMD enrichment of PpsA and PpsE over the PM-CW might have been a consequence of these large proteins sedimenting further in a density gradient than other average-sized proteins due to their large size. However, all five enzymes were identified in the proteome of the immunoprecipitated IMD but not from the mock immunoprecipitation of a wild-type cell lysate. Therefore, our data suggest that these enzymes are truly associated with the IMD. PapA5 transfers two mycocerosates to either a phthiodiolone or a phenolphthiodiolone, producing phthiodiolone dimycocerosate or phenolphthiodiolone dimycocerosate, respectively. These lipids are further modified by sequential reactions of F_420_H_2_-dependent phthiodiolone ketoreductase (fPKR, Rv2951c) and phthiotriol/phenolphthiotriol dimycocerosates methyltransferase (PMT, Rv2952) to become a mature PDIM or phenolphthiocerol dimycocerosate (PPDIM), a precursor of PGL (47–50). Comparative proteomic analysis showed that both fPKR and PMT are enriched in the IMD, and fPKR was also detected in the immunoprecipitated IMD. We attempted to express fPKR with C-terminal mNeonGreen-HA or HA tag but could not detect the protein production by immunoblotting. fPKR has a predicted molecular weight of 41.3 kDa and has no predicted transmembrane domains. Therefore, it is likely that fPKR peripherally associates with the IMD, but further validations are needed.

Finally, we attempted to produce epitope-tagged glycosyltransferases and methyltransferases that are involved in the maturation of PPDIM to PGL. Two methyltransferases (Rv2955c and Rv2959c) and a fucosyltransferase (Rv2957) were found in the IMD proteomes. While we could not detect stable production of epitope-tagged Rv2959c, we were able to produce Rv2955c and Rv2957 with C-terminal HA tag. Both proteins were found to be enriched in the IMD and absent in the PM-CW, confirming the proteomic analysis. Rv2955c was more widely distributed into lighter density regions (Fractions 2–3), which is a feature observed for some *M. smegmatis* IMD proteins as well (e.g., MurG, ThiD) (27, 36). The biochemically validated IMD association of two enzymes involved in the final maturation step of PGL synthesis, combined with other enzymes found in the IMD proteome, suggests that the PDIM/PGL biosynthesis takes place in the IMD.

In sum, we conclude that the IMD is a conserved membrane domain of mycobacteria enriched in lipid biosynthetic enzymes.

## MATERIALS AND METHODS

### Cell cultures

*M. tuberculosis* mc^2^6230 Δ*RD1/panCD* strain was used throughout this study (51). It was grown in Middlebrook 7H9 broth or Middlebrook 7H10 agar supplemented with OADC (final concentrations: 190 µM oleic acid, 0.5% [wt/vol] bovine serum albumin, 11.1 mM dextrose, 13.9 mM NaCl, 0.0004% [wt/vol] catalase), 0.05% Tween 80, and 50 µg/mL pantothenic acid. When required, the medium was supplemented with 100 µg/mL hygromycin B (Wako), 20 µg/mL kanamycin sulfate (MP Biochemicals), 50 µg/mL streptomycin, or 5% sucrose.

### Density gradient fractionation and protein analysis

Log phase cells (OD_600_ = 0.5–1.0) were lysed, and the lysate was fractionated by sucrose density gradient as described previously (16). The protein concentration for each fraction was determined by bicinchoninic acid (BCA) assay (Pierce). Sucrose density of each fraction was determined by a refractometer (ATAGO). The IMD and PM-CW regions were defined as the density region corresponding to 1.08–1.11 g/mL and 1.13–1.17 g/mL, respectively, and fractions that fall into this density range were indicated in each figure. SDS-PAGE and immunoblotting of the sucrose gradient fractionation were performed using equal volume of each fraction as described before (16). Anti-MptC antibody was previously raised (52) and anti-HA antibody was purchased from Sigma-Aldrich.

### Cell-free radiolabeling assay

To determine PPM biosynthesis activities, sucrose density fractions were incubated with GDP-[2-^3^H]mannose (American Radiolabeled Chemicals, 20 Ci/mmol) as described before (17), except that the time of incubation was extended to 1 h. The lipids were extracted, purified, and resolved on high-performance thin layer chromatography (HPTLC) silica gel using chloroform/methanol/13 M ammonia/1 M ammonium acetate/water (180:140:9:9:23, vol/vol/vol/vol/vol) as a solvent and visualized by fluorography using En^3^Hance.

### Lipid analysis

Lipids were extracted from each gradient fraction as described (16) and analyzed by HPTLC as described above. Phospholipids and PIMs were detected by molybdenum blue and orcinol staining, respectively. Additionally, PE was quantified by positive ion LC-MS. Lipid extracts from density gradient fractions were purified by chloroform/methanol extraction followed by butanol/water partitioning. Each extract was spiked with an equal amount of PI C8:0, a non-native lipid standard for normalizing LC-MS peak intensities across samples. Lipid extracts were then dried and resuspended in a mobile phase consisting of 10 mM ammonium carbonate/118.4 mM ammonium hydroxide in acetonitrile-water (60.2:39.8, vol/vol) before being passed through a HILIC liquid chromatography column (Shiseido, Catalog # 93155 type 3 µm) and analyzed by mass spectrometry on a Thermo Orbitrap Fusion mass spectrometer. Peak areas for the phospholipids PE 34:1 and PE 35:0 were normalized to the peak area of PI 8:0 in each fraction and the percentage of the PE 34:1 and PE 35:0 measured across all 12 fractions was compared.

### Negative staining EM

Samples of pooled sucrose gradient fractions of the IMD and the PM-CW were pelleted at 100,000 × *g* for 60 min on TLA100.2 rotor (Beckman), and washed with HES buffer (25 mM HEPES-HCl, pH 7.4, 2 mM EGTA, 150 mM NaCl) twice. The samples were prepared for EM observation as described (16).

### Proteome preparation and analysis

Pooled IMD and PM-CW fractions were pelleted and washed, as described above, in biological triplicates. The protein samples were then separated on a 12% SDS-PAGE gel for a short distance, and the gel slice containing the entire protein bands was subjected to in-gel trypsin digestion. The peptides were analyzed by nano-LC ESI MS on an Orbitrap mass spectrometer (Thermo Scientific Q Exactive) as described (16). The data were analyzed and annotated using Mycobrowser (https://mycobrowser.epfl.ch/). Proteins with a minimum of twofold enrichment (*P* < 0.05, determined by Mann-Whitney Test) in either the IMD or the PM-CW were considered to be part of the respective proteome. Functional annotation was performed using DAVID Bioinformatics Resources as described (16).

The immunoprecipitated IMD proteome was analyzed using the IMD fractions from cells expressing PyrD-HA, using wild-type as a negative control. Immunoprecipitation using HA-agarose beads (Pierce), proteome analysis, and functional annotation analysis were as described (16).

### Construction of plasmids

#### pMUM036

To create an expression vector for PyrD (Rv2139), which is C-terminally tagged with an HA epitope, the *pyrD* gene was amplified with primers A161/A162 from *M. tuberculosis* genomic DNA (see Table S1 at https://doi.org/10.5281/zenodo.17547108). pMUM012 (16) was digested with EcoRV and ScaI, and the vector backbone was blunt-end-ligated with the PCR product.

#### pMUM145

To create a knock-in strain, in which an epitope-tagged PyrD is expressed from the endogenous locus, the upstream and downstream of the *pyrD* gene were amplified using primers A536/A537 and A538/A539, respectively (Table S1). The PCR products were digested with Van91I. The two fragments were then ligated into Van91I-digested pCOM1 as previously described (16) to create an intermediate vector, pMUM133. Using pMV261-CO-TagRFP as a template, tagRFP was amplified and HA tag was added using primers A557/A558. The PCR product and pMUM133 were then digested with VspI and BspT1 and ligated to create a knock-in vector to insert a gene encoding 2×HA-tagRFP at the 3′ end of the *pyrD* gene.

#### pMUM215

To create expression vector for C-terminally mNeonGreen-HA-tagged Psd (Rv0437c), *psd* was cloned by PCR using primers A630/A631 (Table S1). In a separate experiment, a gene encoding mNeonGreen was amplified from pMUM044, an unpublished plasmid which carries the same *mNeonGreen* gene as our published plasmid pMUM072 (16), using primers A483/A484. The PCR product was blunt-end-ligated with ScaI-digested pMUM098 (19) to create pMUM111. The PCR product carrying *psd* and pMUM111 was digested with NdeI and BspTI and ligated to create the final expression vector.

#### pMUM222

To create an expression vector for GlfT2 (Rv3808c) with C-terminal mNeonGreen-HA epitope tag, the *glfT2* gene was amplified with primers A628/A629 (Table S1). The PCR product carrying *glfT2* and pMUM111 was digested with NdeI and BspTI and ligated to create the final expression vector.

#### pMUM257

We first created an expression vector for Rv2955c with C-terminal mNeonGreen-HA epitope tag. The *rv2955c* gene was amplified with primers A849/A850 (Table S1). Both the PCR product carrying *rv2955c* and pMUM235, which is a derivative of pMUM111, were digested with NdeI and BspTI and ligated to create the Rv2955c-mNeonGreen-HA expression vector (pMUM245). To remove mNeonGreen, we digested pMUM245 with PacI and AflII, blunt-ended the digested plasmid using T4 DNA polymerase, and ligated, resulting in pMUM257, the Rv2955c-HA expression vector.

#### pMUM258

As in pMUM257, we first created an expression vector for Rv2957 with C-terminal mNeonGreen-HA epitope tag. The *rv2957* gene was amplified with primers A851/A852 (Table S1). The Rv2957-HA expression vector, pMUM246, and the Rv2957-HA expression vector, pMUM258, were created following the identical procedure described for pMUM257.

Plasmid constructs were electroporated into *Mycobacterium tuberculosis* mc^2^6230 Δ*RD1/panCD* for integration and homologous recombination as previously described (16).

### Fluorescence microscopy

Standard fluorescence microscopy was performed as described (27). To distinguish between cells with or without septa, cells were labeled with the peptidoglycan probe HADA (Tocris Bioscience, Bristol, UK). *M. tuberculosis* cells expressing GlfT2-mNeonGreen-HA were grown to mid-log (OD_600_ = 0.5) and then labeled with 500 µM HADA for 2 h. Labeled cells were washed twice in PBS, spotted onto a 1% agar pad slide and covered with a glass coverslip sealed to the slide with nail polish. Live cells were then imaged with a Nikon Eclipse E600 with a 100× objective lens. mNeonGreen fluorescence profiles along cell lengths were calculated with Oufti (5, 36, 53) and sorted based on the presence or absence of HADA-labeled septa. mNeonGreen fluorescence profiles of septated and non-septated populations were aligned by their brightest pole, normalized by cell length, and then averaged using a custom Python script. Cell polarity of individual cells was calculated from fluorescence profiles using another custom python script. We defined the pole as the brighter of the two 10-pixel (0.74 µm) regions at each end of the cell. Polarity values were calculated as the mean fluorescence of the bright pole region divided by the mean fluorescence value of the whole cell excluding the bright pole region. To prevent biasing of polarity values due to sidewall fluorescence below the detection limit, cells with low fluorescence (cells containing fluorescence intensity values = 0) were removed from our analysis. For SIM, images were acquired by Nikon Eclipse Ti N-SIM E microscope equipped with a Hamamatsu Orca Flash 4.0 camera (numerical aperture, 1.49) as described before (12) and reconstructed on NIS Elements imaging software.

## Data Availability

The mass spectrometry proteomics data have been deposited to the ProteomeXchange Consortium via the PRIDE (54) partner repository with the data set identifier PXD069609 (https://doi.org/10.6019/PXD069609).
